# The long-term consequences of antibiotic therapy: Role of colonic short-chain fatty acids (SCFA) system and intestinal barrier integrity

**DOI:** 10.1371/journal.pone.0220642

**Published:** 2019-08-22

**Authors:** Yuliia Holota, Taisa Dovbynchuk, Izumi Kaji, Igor Vareniuk, Natalia Dzyubenko, Tetiana Chervinska, Liudmyla Zakordonets, Viktoria Stetska, Liudmyla Ostapchenko, Tetiana Serhiychuk, Ganna Tolstanova

**Affiliations:** 1 Taras Shevchenko National University of Kyiv, Kyiv, Ukraine; 2 UCLA/CURE West LA VA Medical Center, Los Angeles, California, United States of America; 3 Bogomolets National Medical University, Kyiv, Ukraine; Southern Illinois University School of Medicine, UNITED STATES

## Abstract

Epidemiological studies revealed that antibiotics exposure increases a risk of inflammatory bowel diseases (IBD) development. It remained largely unknown how antibiotic-induced dysbiosis confers the risk for enhanced inflammatory response. The aim of the present study was to test the hypothesis that SCFAs, their receptors and transporters mediate the antibiotic long-term effects on the functional state of colonic mucosa and susceptibility to the experimental colitis. Male Wistar rats were treated daily for 14 days with antibiotic ceftriaxone (300 mg/kg, i.m.) or vehicle; euthanized by CO_2_ inhalation followed by cervical dislocation in 1, 14 or 56 days after antibiotic withdrawal. We found increased cecum weight and sustained changes in microbiota composition after ceftriaxone treatment with increased number of conditionally pathogenic enterobacteria, *E*. *coli*, *Clostridium*, *Staphylococcus spp*. and hemolytic bacteria even at 56 days after antibiotic withdrawal. The concentration of SCFAs was decreased after ceftriaxone withdrawal. We found decreased immunoreactivity of the FFA2, FFA3 receptors, SMCT1 and increased MCT1 & MCT4 transporters of SCFAs in colon mucosa. These changes evoked a significant shift in colonic mucosal homeostasis: the disturbance of oxidant-antioxidant balance; activation of redox-sensitive transcription factor HIF1α and ERK1/2 MAP kinase; increased colonic epithelial permeability and bacterial translocation to blood; morphological remodeling of the colonic tissue. Ceftriaxone pretreatment significantly reinforced inflammation during experimental colitis 56 days after ceftriaxone withdrawal, which was confirmed by increased histopathology of colitis, Goblet cell dysfunction, colonic dilatation and wall thickening, and increased serum levels of inflammatory cytokines (TNF-α and IL-10). Since the recognition of the importance of microbiota metabolic activity rather than their composition in the development of inflammatory disorders, e.g. IBD, the present study is the first report on the role of the SCFA system in the long lasting side effects of antibiotic treatment and its implication in IBD development.

## Introduction

Recent studies revealed the essential role of microbial metabolites, their receptors and transporters for the host immune response [1–2] and energy homeostasis [3–4]. Among the bacterial metabolites a major component constitutes short-chain fatty acids (SCFA), which are defined as groups of fatty acids having fewer than six carbons, including acetic (C2), propionic (C3), butyric (C4) and valeric (C5) acids. SCFAs have long been known to exert beneficial effects against intestinal inflammation and protecting intestinal epithelial integrity. However, the molecular targets for these bacterial metabolites have been identified only recently.

Extracellular actions of SCFAs occur, in part, through FFA2 and FFA3 G-protein coupled receptors [5–7]. Loss of these receptors has been associated with dysregulated inflammatory responses to different immunologic challenges [8], which suggest that FFA2 and FFA3 are crucial regulators of intestinal inflammation and epithelial barrier function. It has been shown that FFA2 regulates neutrophil chemotaxis [9–10], recruitment of inflammatory mediators [11], regulatory T cells (Treg) development [12] and activates the inflammasome pathway in colonic epithelium [13]. FFA2 on dendritic cells can also promote intestinal IgA production, providing additional route of intestinal epithelium protection against pathogenic microbes [14]. FFA3, unlike FFA2, seems to have a more restricted role in the gut’s barrier function. Expressing on the nonimmune cell population of the gut FFA3 has been shown to confer protection in infectious colitis model mainly by enhancing expression of cytokines and chemokines through the MEK-ERK (mitogen-activated protein/extracellular signal regulated kinase kinase) pathway [8].

Besides activation of receptors presented on the cell surface, SCFAs also have intracellular actions (e.g., histone deacetylase inhibition, metabolic regulation, etc.), which obviously require their entry into colonic epithelial cells. SCFAs produced by the microbiota in the cecum and colon can be found in hepatic, portal, and peripheral blood. Several transport systems operate in cellular uptake of SCFAs such as H^+^-coupled transporters (MCT1 and MCT4) and Na^+^-coupled transporters (SMCT1 and SMCT) [15]. SCFAs transport deficiency is closely associated with pathological conditions such as ulcerative colitis [16] and colon cancer [17] that confirms the role of SCFAs transporters as the major determinants of the beneficial effects of SCFA on the host.

There is increasing concern that antibiotic exposure has long-term consequences [18–20]. Epidemiological studies revealed that antibiotic-induced disruption of the gut microbiota increased the risk of multiple disorders [21–23] including inflammatory bowel diseases (IBD) [24–25].

Alterations in the intestinal microbial composition have long been associated with chronic inflammation. Howether, remained largely unknown how antibiotic-induced dysbiosis confers a risk for enhanced inflammatory responses. Knoop et al. [26] showed that oral antibiotics induce the translocation of live native commensal bacteria across the colonic epithelium, promoting inflammatory responses, and predisposing to a more severe disease in response to coincident injury in mice. Disruption of the microbiota with metronidazole increased the inflammatory tone of the intestine characterized by a weakened mucosal barrier contributing to the exacerbated severity of *C*. *rodentium*-induced colitis [27]. These studies confirm that alterations of the intestinal microbiota composition can significantly affect host immunity and the course of mucosal inflammation.

Gut bacteria respond to antibiotic therapy by activating systems of avoiding the antimicrobial effects, while ‘presumptively’ attenuating their overall energetic and metabolic status. It was shown that β-lactam antibiotics affect the most variable metabolic functions of the microbiota [28], including the decrease of SCFAs production [29–32]. There is lack of data on how the host organism responds to these changes long-after antibiotic withdrawal. The aim of the present study is to test the hypothesis that SCFAs, their receptors and transporters mediate the antibiotic long-term effects on the functional state of colonic mucosa and susceptibility to the experimental colitis.

## Materials and methods

### Animals

Male Wistar rats (140–160 g, n = 184) were bred and housed in a conventional animal facility at the ESC “Institute of Biology and Medicine” Taras Shevchenko National University of Kyiv (Kyiv, Ukraine). Animals were kept under controlled conditions of illumination (12-hour light/dark cycles), temperature (21–23°C), and humidity (30–35%). All animals had unlimited access to animal chow and tap water throughout the study. To normalize gut microbiota, rats from all groups were kept in the same room, given free access to standardized rodent diet and maintained by the same personnel.

The study was carried out in strict accordance with the Institutional Animal Care and Use Guidelines. The study design was approved by the Bioethical Committee of the ESC “Institute of Biology and Medicine” of Taras Shevchenko National University of Kyiv (Protocol Number 8 issued on Nov 2, 2015).

### Study design

#### Antibiotic treatment

Ceftriaxone (Ind. Stock Company Darnytsya, Ukraine) was injected intramuscularly at a dose of 300 mg/kg, daily for 14 days. Rat equivalent dose was calculated based on body surface area by multiplying the human dose (50 mg/kg) by the K_m_ value (6 for rats) [33]. Control rats were treated with sterile water (0.1 ml/rat, i.m.). Body weight and lethargy were controlled throughout experiment. Stool samples for microbiota and SCFA analysis were collected on the 1^st^, 14^th^ or 56^th^ day after ceftriaxone withdrawal right before autopsy. During autopsy rats were euthanized by CO_2_ inhalation with further cervical dislocation. Colon from anus to ileum was removed. Cecum was cut and weighed. The two cm piece of colon at the distance of 2 cm from the anus was cut and embedded in 10% buffered formalin following paraffin for further immunohistochemical analysis. Rest of the colon was cut along the anti-mesenteric side and thoroughly rinsed in cold sterile PBS. Colon was gently wiped with a paper towel and flattened by mucosa side up on ice. The mucosa was gently scraped by metal spatula from the muscular layer and embedded in liquid nitrogen for further biochemical assays.

#### Iodoacetamide-induced colitis

Experimental colitis was induced in control and antibiotic-treated rats by the sulfhydryl alkylator iodoacetamide [34] on the 56^th^ day after ceftriaxone withdrawal. Briefly, rats were administered once by rectal enema (7 cm from the anus) via rubber catheter either 0.1 ml of 3% iodoacetamide (Sigma, USA) dissolved in 1% methylcellulose (Sigma, USA) or the vehicle 0.1 ml of 1% methylcellulose. Thereby animals were divided into 4 treatment groups: control groups, treated with sterile water for 14 days and either 1% methylcellulose enema (Control) or 3% iodoacetamide enema (IA) on the 56^th^ day after sterile water withdrawal; experimental groups, treated with ceftriaxone 300 mg/kg for 14 days and either 1% methylcellulose enema (Ceftriaxone) or 3% iodoacetamide enema (Ceftriaxone + IA) on the 56^th^ day after ceftriaxone withdrawal. Rats were euthanized by CO_2_ inhalation with further cervical dislocation after 6 h after iodoacetamide or methylcellulose enema.

During autopsy, we cut 7 cm of the distal colon from anus. The removed colon was opened longitudinally, rinsed with saline, gently blotted with filter paper, and weighted. Macroscopic colon damage was assessed as described previously [34]. Briefly, colitis severity was scored on a scale of 0–3 (0 –normal; 1 –mucosal erosion; 2 –moderate lesion; 3 –deep lesion). The colonic lesion areas (mm^2^), loss of rugae areas (mm^2^), colonic dilatation (mm), colonic thickness (0 –no change; 1 –mild; 2 –moderate; 3 –severe thickening) and colon wet weight (g/100 g body weight) were assessed.

### Processing of fecal specimens for microbiological analysis

Faecal materials (1g) were sampled during an autopsy and immediately dispersed in 9 ml sterile solution of 0.5% NaCl. Serial dilutions (10^−1^, 10^−3^, 10^−5^, 10^−7^, and 10^−8^) of each sample were prepared. Selective media were used to study the bacterial subpopulations: Bifidobacterium Agar (HiMedia, India) for *Bifidobacterium*; MRS Agar (HiMedia, India) for lactobacilli group (subsequently called *Lactobacillus*); Iron Sulphite Agar (HiMedia, India) for *Clostridium*, Mannitol Salt agar (HiMedia, India) for *Staphylococcus* (mannitol-fermenting colonies were considered as *Staphylococcus aureus*, while mannitol non-fermenting colonies were considered as *Staphylococcus spp*.); Simmons Citrate Agar (HiMedia, India) for citrate-fermenting conditionally pathogenic enterobacteria; Blood Agar (HiMedia, India) with 5% w/v sterile defibrinated sheep blood (Hemostat Laboratories, USA) for hemolytic bacteria. After incubation for 24–48 hours at 37°C, plates were examined and colonies were counted. Identification of isolates was done by examination of colony color and morphology on primary isolation plates and cell morphology in gram-stained slides.

A presumptive test for members of the coliform group was done on Endo Agar (HiMedia, India). Both lactose-fermenting (pink) and lactose non-fermenting (colorless) colonies from Endo Agar after incubation at 37°C for 24 hours were transferred on the Triple Sugar-Iron Agar Medium (HiMedia, India). Lactose-fermenting, gas-forming, H_2_S negative colonies were considered as *Escherichia coli* with normal enzymatic properties (subsequently called *E*. *coli* lactose-fermenting). Lactose non-fermenting, gas-forming, H_2_S negative colonies were considered as *Escherichia coli* with altered enzymatic properties (subsequently called *E*. *coli* lactose non-fermenting). After identification of microorganisms, which grew as single colonies in the dilutions, the quantitative composition was determined. The number of fecal microorganisms was calculated as lg of colony forming unit per 1 g of feces (lg CFU/g).

### Gastrointestinal (GI) transit assay

Carmine red, which cannot be absorbed from the lumen of the gut, was used to study the total GI transit time [35]. Rats were orally gavaged with 0.5 ml aqueous solution of 3% carmine red (Sigma Aldrich) and placed in a new cage with no bedding. The time at which gavage took place was recorded as T0. Starting at 120 min post-gavage, rats were monitored every 10 min for production of a red fecal pellet. Total GI transit time was considered as the interval between T0 and the time of the first observance of carmine red in stool [36].

### Fecal SCFA analysis

Fecal samples (1 g) were homogenized in 2 ml of 0.02N HCl and kept at room temperature for extraction during 30 min in air-tight containers to prevent the loss of volatile SCFAs. Samples were then centrifuged for 10 min at 11000 g and 300 μl of supernatants were transferred into an autosampler vial for GC-MS analysis. 0.05% 4-methylvaleric acid (Sigma-Aldrich, Germany) was used as internal standard.

Gas chromatographic (GC) analysis was carried out using an Agilent 6890N GC system (Agilent Technologies, USA) equipped with an automatic liquid sampler (7683B, Agilent Technologies, USA). Separation was performed using a fused-silica capillary column with a free fatty acid phase DB_FFAP 0.25 μm × 0.25 mm × 30 m (Agilent Technologies Inc., USA). Helium was supplied as the carrier gas at a flow rate of 1 mL/min. Temperature of the injection port was 250°C. The injected sample volume for GC analysis was 1 μL with split ratio 1:20. The initial oven temperature was 100°C, maintained for 5 min, raised to 190°C at 10°C/min. The run time for each sample was 16 min. A single quadrupole mass spectrometer (Agilent, 5973 inert MSD) was used for detection of SCFAs. Data handling was carried out with Chem Station Data Analysis D.01.02.16 software.

The SCFAs were identified on chromatograms by their specific retention times of standard SCFAs mixture of acetic, propionic, *i*-butyric, *n*-butyric, *n*-valeric, *i*-valeric, *n*-caproic acids (Sigma-Aldrich, Germany) under the above GC conditions. To quantify the peak area in terms of concentration, the peak area was plotted against the peak area and concentration of the internal standard. Absolute quantities of SCFAs were normalized to sample mass (μmol/g feces).

### Immunohistochemistry

Immunostaining was performed using paraffin-embedded 5-μm-thick intestinal sections. Sections were deparaffinized, hydrated. Antigen retrieval was performed by incubating the sections for 30 min at 98°C in citrate buffer, pH 6. The activity of endogenous peroxidase was blocked by incubating sections with 0.3% v/v hydrogen peroxide for 3 min. Sections were then washed with 0.05M Tris-buffered saline containing 0.15M NaCl and 0.05% v/v Tween 20, pH 7.6. Colon sections were then incubated with primary antibodies: rabbit anti-FFA2 (1:300; Frontier Institute Co. Ltd., Ishikari, Japan), rabbit anti-FFA3 (1:400; Frontier Institute), rabbit anti-MCT1 (1:200; Frontier Institute), rabbit anti-MCT4 (1:200; Frontier Institute) or rabbit anti-SMCT1 (RY1617, 1:1,000) (Iwanaga et al. 2006 Biomedical Research) at room temperature for 30 min or overnight (for MCTs and SMCT1). For FFA2 and FFA3 immunoreactivity detection sections were then washed and incubated with DakoPolyclonal Swine Anti-Rabbit HRP secondary antibody (1:100) at room temperature for 30 min. Histological signal was developed using 3,3’-diaminobenzidine in a chromogen solution before counterstaining the sections with Mayer’s hematoxylin. Sections were dehydrated and mounted using the mounting medium (Dako, Victoria, Australia) and then examined microscopically for positively stained cells. For MCTs and SMCT1 detection, sections were incubated with Cy3-conjugated donkey anti-rabbit IgG (1:400; Jackson ImmunoResearch, West Grove, PA, USA) and SYTO 13 (SYTOX, Invitrogen, Carlsbad, CA, USA), and observed under a confocal laser scanning microscopy (Fluoview, Olympus, Tokyo, Japan).

### Western blot analysis

Colonic mucosa samples were homogenized in the lysis buffer containing 50 mM Tris-HCl (pH 7.4), 150 mM NaCl, 1% sodium deoxycholate, 0,1% SDS, 1% Triton-100, 10 mM sodium orthovanadate (Na_3_VO_4_) and complete protease inhibitor cocktail (Roche, Mannheim, Germany) and centrifuged at 14,000 rpm for 15 min at 4°C. The total concentration of proteins was measured with “Bio-Rad protein assay” (Bio-Rad, USA). After mixing with 2x Laemmli sample buffer (Sigma-Aldrich, USA) 1:1 v/v and heating at 95°C for 5 min samples (100 μg of protein) were run on 12% (for Erk1/2, p38, FFA2, FFA3 proteins) and 8% (for Hif1α) SDS-PAGE gels and transferred onto the Hybond-ECL nitrocellulose membrane (Amersham Biosciences, USA) according to a standard protocol of Bio-Rad Company. Then, blots were blocked with 1% dry milk and 1% BSA in TBST (10 mM Tris-HCl pH 7.4, 150 mM NaCl, and 0.05% Tween-20) at 4°C overnight. Rabbit polyclonal anti-Erk1/2 (1:1000, Santa-Cruz, USA), mouse monoclonal anti-pErk (1:1000, Santa-Cruz, USA), rabbit polyclonal anti-p38α/β (1:200, Santa-Cruz, USA), rabbit polyclonal anti-p-p38 (1:200, Santa-Cruz, USA), mouse monoclonal anti-Hif-1α (1:500, Novus Biologicals, USA), rabbit polyclonal anti-FFA2 (1:500, Santa-Cruz, USA), rabbit polyclonal anti-FFA3 (1:300, Santa-Cruz, USA) and mouse monoclonal anti-𝛽-actin antibodies (1:1000) were used as a primary antibody with incubation time of 1 hour at room temperature, washing three times with TBST followed by 1-hour incubation by donkey horseradish peroxidase-conjugate anti-rabbit IgG (1:2500, Santa-Cruz, USA), or goat anti-mouse IgG-HRP (1:2500, Santa-Cruz, USA). The light signal was captured on an X-ray film. Densitometric analysis of protein bands was performed using LI-COR Image Studio Software Lite Ver. 5.2. The level of each protein was normalized against 𝛽-actin band intensity.

### Catalase and superoxide dismutase activity assay

Catalase activity in colonic mucosa was assessed colorimetrically in a reaction with 0.03% H_2_O_2_ solution. The reaction was stopped by the molybdate ammonium (Alfarus, Ukraine) and measurement was taken at a wavelength of 410 nm. The activity of superoxide dismutase (SOD) was determined by in-gel activity assay [37–38]. Colonic mucosa samples were electrophoresed in 10% native-polyacrylamide gel at 40 mA at 4°C. After electrophoretic separation, the gel was incubated for 20 min in 50 mM phosphate buffer solution (pH 7.8) containing 0.25 mM NBT, 1 mM EDTA, 28 mM TEMED, and 0.028 mM of riboflavin (Sigma-Aldrich, Germany). Gel was developed by illumination. Achromatic bands on the light-blue background of the gel indicated the presence of SOD. Densitometry was performed using LI-COR Image Studio Software Lite Ver. 5.2.

### Measurement of malondialdehyde (MDA) level in colonic mucosa

The level of thiobarbituric acid-reactive substances, such as malondialdehyde (MDA) was used as a marker for lipid peroxidation in colonic mucosa. Samples were homogenized in 25 mM Tris-HCl (pH 7.4), mixed with 20% trichloroacetic acid (Alfarus, Ukraine) and centrifuged at 3,000 rpm for 15 minutes. Thereafter, 0.5 ml of each supernatant was added to 0.25 ml of 0.8% thiobarbituric acid (Alfarus, Ukraine) and heated for 10 min at 100°C. The absorbance of samples was determined spectrophotometrically at a wavelength of 532 nm against blank reference. Colonic MDA level (mg/g protein) was counted using coefficient of MDA molar extinction 1.56 × 10^5^ M^–1^ cm^–1^.

### Protein thiol (SH) groups assessment

Protein SH groups level in colonic mucosa was determined using Ellman’s reagent [39]. The total level of SH groups in colonic mucosa was measured after sample incubation with Ellman’s reagent (2.6 mM 5,5′-dithiobis-2-nitrobenzoic acid) for 30 min at room temperature. For measuring non-protein SH groups samples were mixed with 10% trichloroacetic acid (Alfarus, Ukraine) and centrifugated at 3,000 rpm for 15 minutes. A supernatant was neutralized with 1M NaOH. Then, samples were incubated with Ellman’s reagent. The absorbance of samples was measured at a wavelength of 412 nm at room temperature against blank reference. Protein SH groups level (mmol/g protein) was calculated using coefficient of trinitrophenol anion molar extinction 14.15 M^–1^ cm^–1^.

### Matrix metalloproteinase (MMP) gelatin zymography

Proteolytic activity of MMP-2 and MMP-9 in colonic mucosa was detected by electrophoretic zymography [40]. In brief, 100 μg of total protein extracted from each colonic mucosa sample was run on 10% SDS-PAGE gel with gelatin (1 mg/ml) at 125 V. MMP activity was restored by removal of SDS by gentle shaking at room temperature in Triton X-100 (2.5%) for 30 min followed by incubation in Zymogram Developing Buffer (50 mM Tris base, 50 mM Tris acid, 0.2 mM NaCl, 5 mM CaCl_2_, and 0.02 mM Brij) at 37°C overnight. Thereafter gels were stained in Coomassie Brilliant Blue (0.25%) in methanol/acetic acid/water (50:10:40, v/v/v), and destained in the same solution without dye. Proteolytic activity was visualized as clear bands of lysis on a blue background of undigested gelatin. The molecular mass of the enzymes was determined by comparison with protein standards (Bio-Rad Laboratories) on the same gel.

### *In Vivo* assessment of colonic permeability

Colonic permeability was assessed by the Evans blue permeation method [41]. Briefly, rats were anesthetized with urethane (1.1 g/kg, i.p.), laparotomy was performed and the portal vein was catheterized. The rat colon was ligated and instilled with 1.5% Evans blue solution. Blood samples (0.2 ml, in ice-chilled tubes containing EDTA) were collected every 30 min after Evans blue intracolonic injection for the 1.5-h period. An equal volume of saline was reinjected after each blood sample withdrawal. Plasma concentration of Evans blue was measured by dual-wavelength spectrophotometry. The absorbance was read at 620 nm with correction for any contaminating heme pigments with the following formula: corrected absorbance at 620 nm = actual absorbance at 620 nm − [1.426 (absorbance at 740 nm) + 0.03]. The increase in colonic permeability was determined by the difference in blood Evans blue level at 30 and 60 minutes after intracolonic injection.

### Assessment of bacterial translocation

To determine the level of bacterial translocation to the blood, the rats were anesthetized with urethane (1.1 g/kg, i.p.. Sigma-Aldrich, Germany). After aseptic laparotomy, a sterile catheter was inserted into the portal vein and 1 ml of blood was collected and diluted into 9 ml of sterile saline solution. Enriched solutions were quantitatively plated onto agar media containing 5% sheep blood (HiMedia, India) and incubated over-night at 37°C. The number of microorganisms was calculated as lg of colony forming unit per 1 ml of blood (lg CFU/ml).

### Histological examination

Colonic sections were fixed for more than 12 hours in 10% formalin solution, or in metha-Carnoy solution containing 60% absolute methanol, 30% chloroform, 10% glacial acetic acid. After fixation the samples were dehydrated, embedded in paraffin with a vertical orientation and cut into 5-μm-thick sections. Tissue sections (after fixation in 10% formalin solution) were stained with a hematoxyline and eosine (H&E) using standard techniques. For the morphometric analysis, the digital microphotographs of stained colon sections were taken at a magnification of ×100 or ×400 using a computer-assisted image analyzing system. The thickness of mucosa (μm), depth of crypts (μm), the height of enterocytes (μm) and the area of enterocytes nucleus (μm^2^) were assessed using Image J software. Alcian blue staining (after fixation in metha-Carnoy solution) was performed using standard procedures for visualization of mucous-secreting goblet cells in the colon with Alcian blue pH 2.5 kit (LLC “BioVitrum”, Russia); cellular area of goblet cell (μm^2^) and goblet cells number per crypts area (mm^2^) were assessed.

### Determination of TNF-α and IL-10 serum levels

Blood samples were collected by a cardiac puncture in rats. The collected blood was left for 15 min at 37°C to clot, followed by centrifugation at 3500 g for 15 min. The serum was transferred to clean tubes and stored at −20°C for further analysis. Serum interleukin-10 (IL-10) and tumor necrosis factor-α (TNF-α) levels were measured by enzyme immunoassay kits for the quantitative determination of IL-10 (Vector-Best, Russia) and TNF-α in serum (Vector-Best, Russia), respectively. All measurements were performed in duplicate.

### Statistical analysis

Data are presented as Mean ± SEM. The statistical significance was determined by the Student’s *t*-test where appropriate. The not normally distributed log transformed counts were compared using non-parametric tests (Mann–Whitney U-test). P-values of less than 0.05 were considered statistically significant. In addition to summary statistics, the data points behind means are available (S1 File).

## Results

### A 2-week ceftriaxone treatment induced a sustainable shift in culturable fecal microbiota

**Fig 1:**
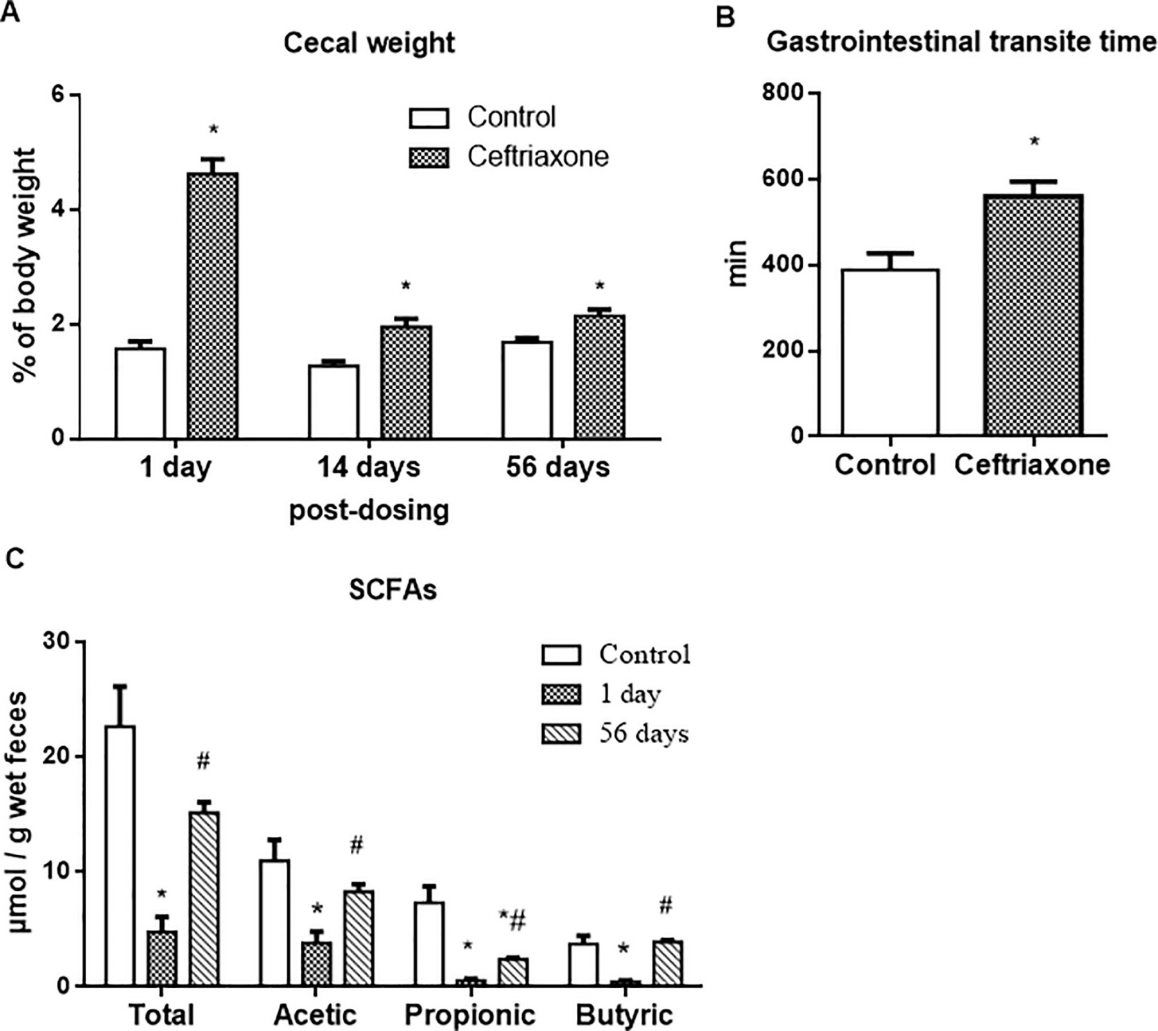
Level of short-chain fatty acids (SCFAs) in rat feces is decreased after ceftriaxone administration (300 mg/kg, i.m., 14 days). (A) Weight of rat cecum at different time points after ceftriaxone withdrawal, n = 18; (B) Gastrointestinal transit time 1 day after ceftriaxone withdrawal, n = 10, data were obtained using carmine red dye; (C) The level of SCFAs in rat feces at different time points after ceftriaxone withdrawal, n = 12, fecal level of SCFAs was examined by gas chromatography; M ± SEM; * P < 0.05 vs. control values; # P < 0.05 vs. one day after ceftriaxone withdrawal.

To determine the long-term consequences of a single antibiotic administration for microbial profile, we treated rats with the broad-spectrum antibiotic ceftriaxone for 2 weeks. This treatment did not affect the rate of rat body weight within all studied period. While at autopsy, we observed the significant enlargement of the cecum in antibiotic treated rats, especially on the 1^st^ day (2.9 fold, P < 0.05), with less profound changes on the 14^th^ (1.5 fold, P < 0.05) and 56^th^ days (1.3 fold, P < 0.05) after antibiotic withdrawal (Fig 1A). The sharply increased cecum weight one day after ceftriaxone withdrawal was accompanied by increased 1.4-fold (P < 0.05) gastrointestinal transit time (Fig 1B).

Since the enlargement of the cecum is the feature of dysbiosis [42], to further confirm these observations we checked on fecal microbiota composition. Fecal microbiota was analyzed at 1, 14 and 56 days after ceftriaxone withdrawal (Table 1). We did not find any significant changes in the number of *Lactobacillus* and *Bifidobacterium* at all studied periods. However, the number of citrate-fermenting conditionally pathogenic enterobacteria and hemolytic bacteria increased sharply. Among the bacteria of the *Staphylococcus* genus, a trend to decrease *Staphylococcus aureus* was observed, while the number of mannitol non-fermenting *Staphylococcus spp*. was increased. Lactose-fermenting *Escherichia coli* was not detected next day after ceftriaxone withdrawal, however, in 14 days their number was not significantly changed *vs* control value. At 56 days after ceftriaxone withdrawal, the number of lactose-fermenting and lactose non-fermenting *E*. *coli*, conditionally pathogenic enterobacteria and *Clostridium* was increased dramatically compared to control values.

**Table 1:** The counts (lg CFU/ g feces) of fecal microorganisms at different time points after 14-day administration of ceftriaxone (300 mg/kg, i.m.) in rats.

Group of microorganisms	lg CFU/g feces			
		*Ceftriaxone*, *after withdrawal*		
	*Control*	*1 day*	*14 days*	*56 days*
*Bifidobacterium*	8. 86 ± 0.24	8.77 ± 0.24	8.13 ± 0.22	8.80 ± 0.15
*Lactobacillus*	8.79 ± 0.16	8.18 ± 0.48	8.76 ± 0.06	9.00 ± 0.21
*Clostridium*	1.94 ± 0.06	1.74 ± 0.19	2.20 ± 0.20	3.18 ± 0.48*^#^
*Escherichia coli* lactose-fermenting	5.21 ± 0.27	0.00*	5.14 ± 0.92	**7.04 ± 0.51**^**#**^
*Escherichia coli* lactose non-fermenting	1.72 ± 0.85	2.22 ± 0.92	2.50 ± 1.40	4.12 ± 0.78*
Citrate-fementing conditionally pathogenic enterobacteria	1.08 ± 0.44	4.10 ± 0.13*	3.96 ± 0.01*	6.02 ± 0.68*^#^
*Staphylococcus aureus*	6.41 ± 0.07	3.95 ± 0.67*	4.42 ± 0.24	6.57 ± 0.48^#^
*Staphylococcus spp*.	1.02 ± 0.08	4.68 ± 0.58*	2.40 ± 0.98	6.69 ± 0.26*^#^
*Hemolytic bacteria*	4.23 ± 0.23	7.63 ± 0.55*	5.06 ± 0.42	6.34 ± 0.48*

* P < 0.05 *vs*. сontrol

# P < 0.05 *vs*. one day after ceftriaxone withdrawal.

Consequently, the parenteral administration of ceftriaxone provoked significant sustainable alterations in the fecal culturable microbiota, which is similar to clinical dysbiosis.

### A 2-week ceftriaxone treatment induced sustainable changes in fecal concentration of SCFAs

Because bacterial strains are very individual markers either in humans or experimental animals, we checked on the microbial metabolites that are more reliable and are even markers relevant to the number of colonic functions. Since SCFAs are the main metabolites of the intestinal microbiota, we determined whether ceftriaxone treatment was associated with long lasting alterations in fecal SCFAs content. To address this, we measured the concentrations of acetic, propionic and butyric acids on the 1^st^ and 56^th^ days after antibiotic withdrawal. On the 1^st^ day, we found a profound decrease of the total SCFA concentration and individually acetic, propionic and butyric acids (P < 0.05) (Fig 1C). At 56 days, we observed partial restoration of SCFAs level, but still concentration of propionic acid were lower than in control samples (P < 0.05).

### A 2-week ceftriaxone treatment changed distribution and levels of SCFAs transporters in rat colon

**Fig 2:**
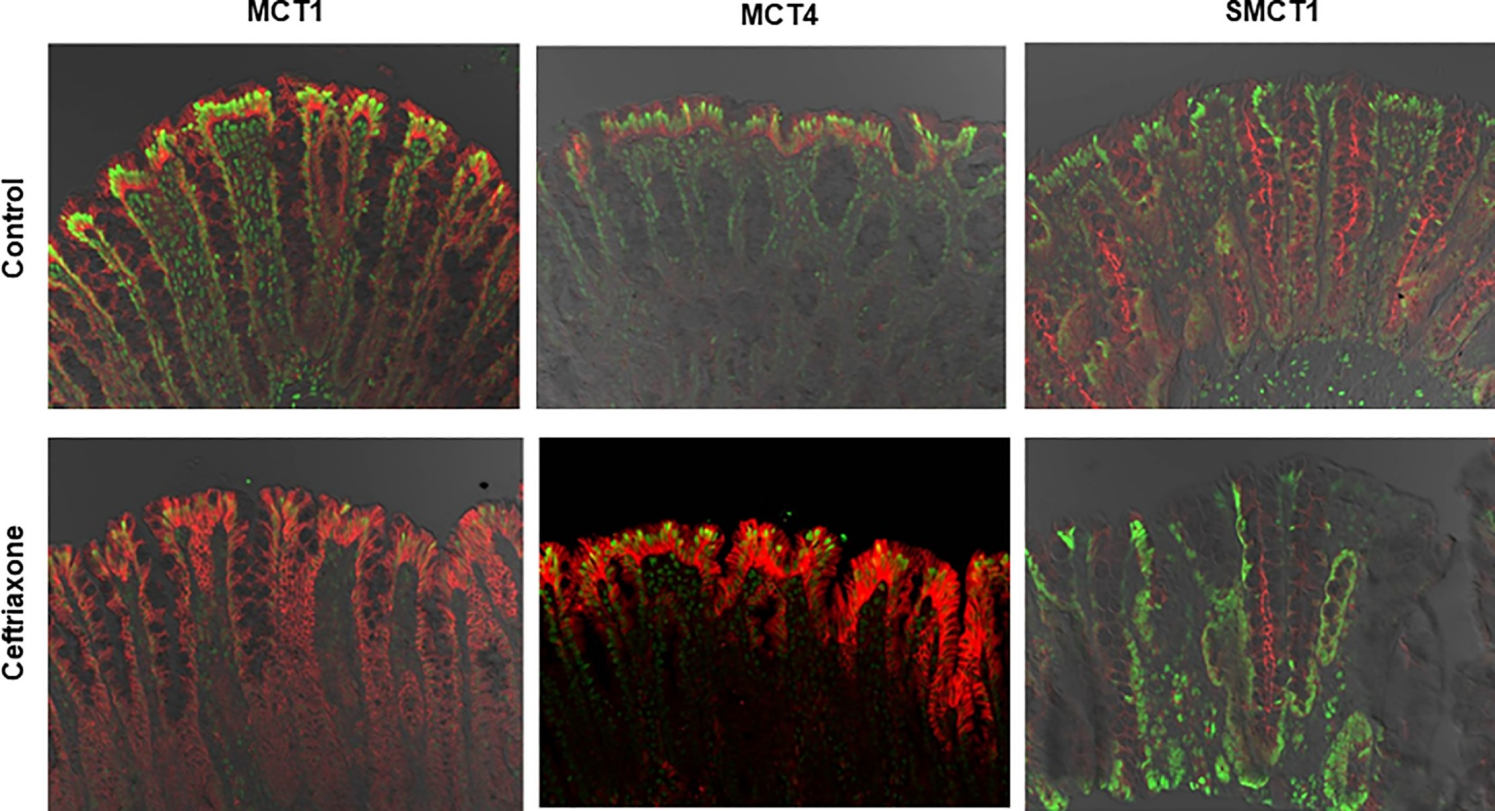
Ceftriaxone administration (300 mg/kg, i.m., 14 days) altered the immunoreactivity (IR) of the SCFA transporters in the rat distal colon. The MCT1-IR was localized on the basolateral membrane of crypt cells, MCT4-IR was on the basolateral membrane of enterocytes lining the upper half of crypts, and SMCT1-IR was on the brush border of enterocytes in control rats. The IR of MCTs was increased next day after ceftriaxone withdrawal, whereas that of SMCT1 was decreased. n = 5; Immune positive staining is shown in red; Nuclei were visualized in green; x400.

In order to elucidate how colonic enterocytes react to antibiotic-associated SCFAs depletion, we examined immunoreactivity of SCFAs transporters. Monocarboxylate transporters (MCT1 and MCT4) immunoreactivity was detected on the epithelial basolateral membrane in the distal colon. MCT4 was localized to the basolateral membrane of epithelial cells lining of the upper half of crypts, whereas MCT1 was ubiquitously detected on the basolateral membrane of crypt cells. Immunoreactivity for sodium-dependent monocarboxylate transporter 1 (SMCT1) was localized on the brush border of enterocytes. The frequency of immunoreactivity of MCT1 and MCT4 was higher at 1 day after ceftriaxone withdrawal, whereas SMCT1 were decreased compared with the control group (Fig 2). Consequently, the colonic epithelial cells of rats alter the expression of SCFA transporters in response to a decrease of SCFAs production by intestinal microbiota.

### A 2-week ceftriaxone treatment changed distribution and expression of SCFAs receptors in rat colon

**Fig 3:**
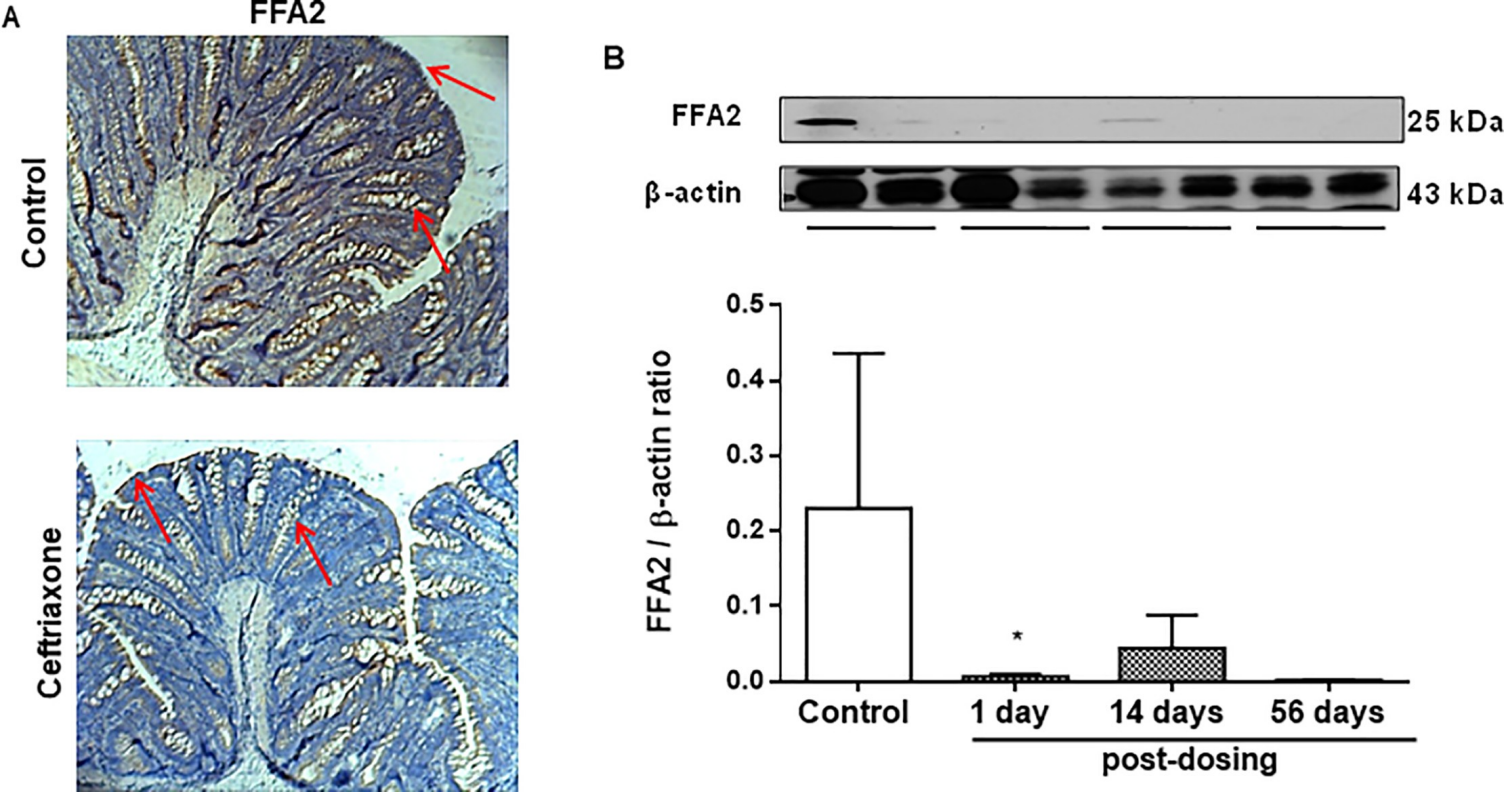
Ceftriaxone administration (300 mg/kg, i.m., 14 days) provokes sustained decrease expression of free fatty acid receptor 2 (FFA2) of SCFAs in rat colon. (A) FFA2 receptors were localized on surface and crypt enterocytes (red arrows) of the rat distal colon; immunoreactivity was decreased one day after antibiotic withdrawal; positive staining–brown, x100, n = 6. (B) FFA2 receptors in rat colonic mucosa at 1, 14 and 56 days after ceftriaxone withdrawal; Western blot; loading control– β-actin; n = 16; M ± SEM; * P < 0.05 *vs*. control values.

**Fig 4:**
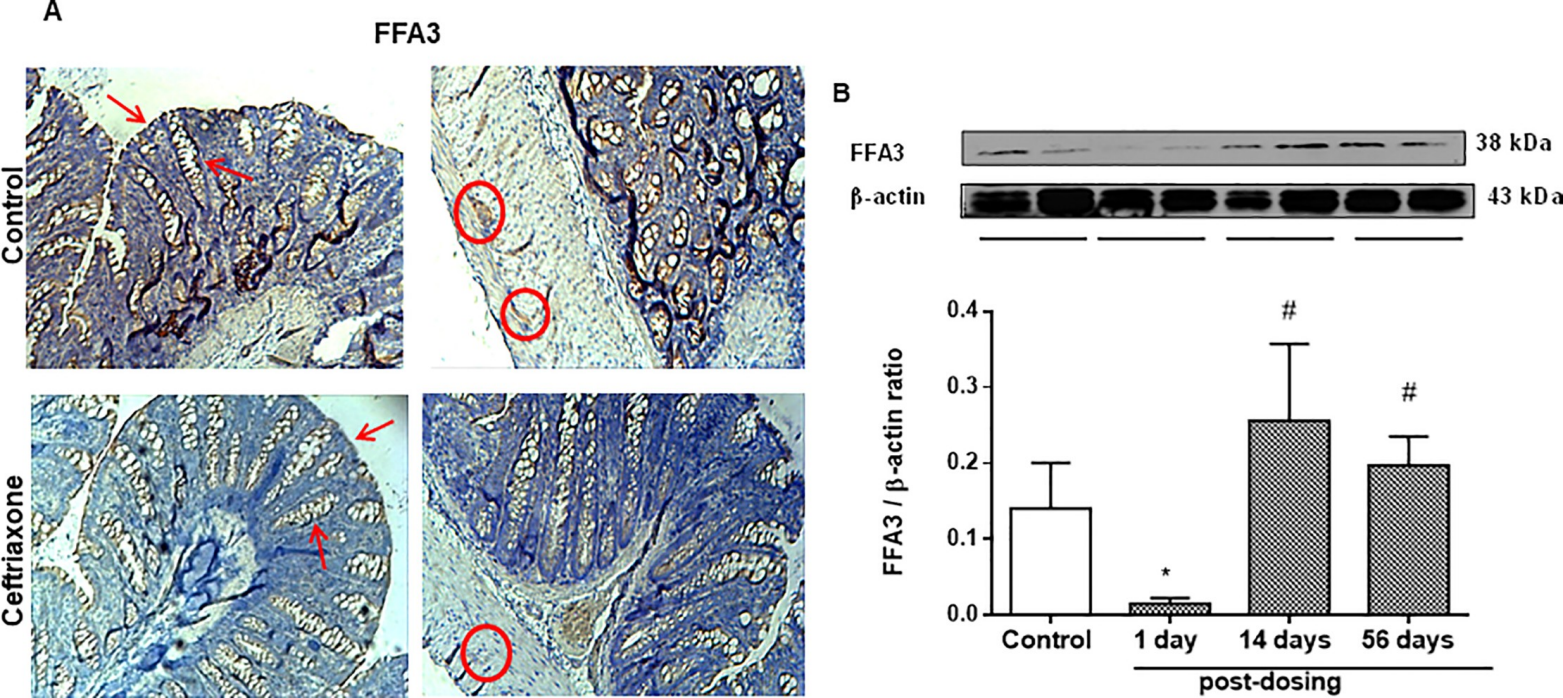
Expression of free fatty acid receptor 3 (FFA3) of SCFAs in rat colon was decreased next day after ceftriaxone administration (300 mg/kg, i.m., 14 days). (A) FFA3 receptors were localized on the surface and crypt enterocytes (red arrows), myenteric ganglia (red rings) of the rat distal colon; immunoreactivity was decreased one day after antibiotic withdrawal; positive staining–brown; x100, n = 6. (B) FFA3 receptors in the rat colonic mucosa at 1, 14 and 56 days after ceftriaxone withdrawal; Western blot; loading control– β-actin; n = 16; M ± SEM; * P < 0.05 *vs*. control values, # P < 0.05 *vs*. one day after ceftriaxone withdrawal.

We found profound expression of both FFA2 and FFA3 receptors in the rat colonic tissue (Figs 3 and 4). FFA2 receptors localized on surface enterocytes and at the crypt bottom. FFA3 receptors localized on the surface enterocytes, goblet cells and neurons of the myenteric ganglia. Next day after ceftriaxone withdrawal we observed significant total reduction in both FFA2 and FFA3 immunoreactivity in the rat colon (Figs 3A and 4A). Western blot analysis revealed sustained decrease in FFA2 levels at 1, 14 and 56 days after ceftriaxone withdrawal *vs*. control group (Fig 3B), while the level of FFA3 receptors decreased 9.3 fold (P <0.05) only on the 1^st^ day, but returned to control levels between 14^th^ and 56^th^ days after ceftriaxone withdrawal (Fig 4B).

Thus, ceftriaxone administration induces sustained changes in the fecal SCFAs production, the level of expression of their receptors and transporters in the rat colon. These data indicate the disturbance of microbiota and macroorganism communication long-after antibiotic therapy that can precede inflammatory bowel disease development.

### A 2-week ceftriaxone treatment disturbed oxidant-antioxidant balance in the rat colonic mucosa

**Fig 5:**
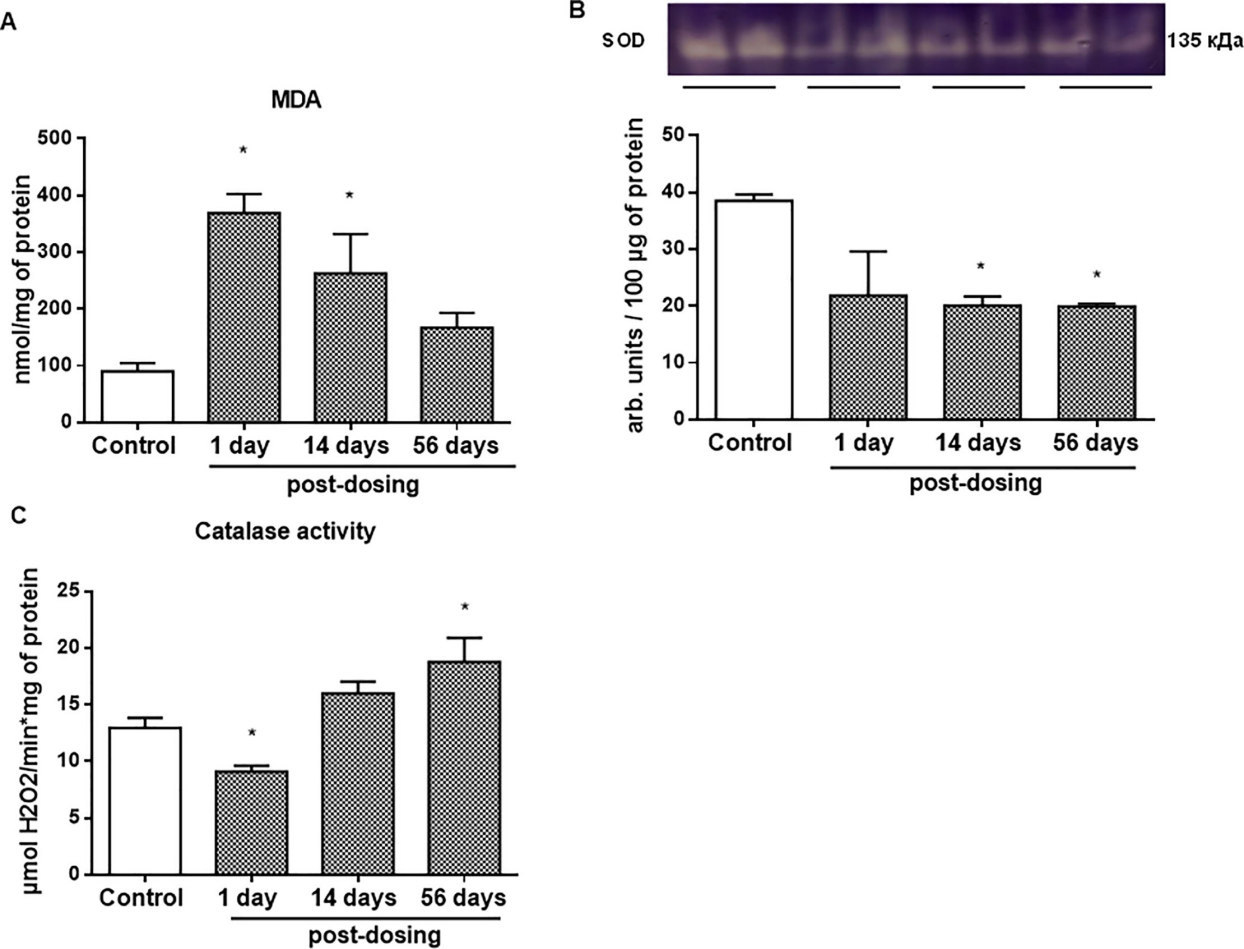
Increased malondialdehyde (MDA) level and altered superoxide dismutase (SOD) and catalase antioxidant enzymes activity after ceftriaxone dosing (300 mg/kg, i.m., 14 days). (A) Level of MDA (the intensity of lipid peroxidation) in colonic mucosa 1, 14, 56 days after ceftriaxone withdrawal. (B) SOD activity in colonic mucosa 1, 14, 56 days after ceftriaxone withdrawal. (C) Catalase activity in colonic mucosa 1, 14, 56 days after ceftriaxone withdrawal. n = 30; M ± SEM; * P < 0.05 *vs*. control values.

We hypothesized that reducing the metabolic activity of the intestinal microbiota after ceftriaxone administration might promote functional changes in colonic mucosa. Colonic mucosal MDA level (the intensity of lipid peroxidation) was increased 6- and 2.5-fold (P < 0.05) 1 and 14 days after ceftriaxone withdrawal, respectively (Fig 5A). These changes were accompanied by an imbalance of antioxidant enzymes activity. SOD activity in the rat colonic mucosa was decreased by 1.9-fold (P < 0.05) between 14^th^ and 56^th^ days after antibiotic withdrawal (Fig 5B). In parallel, catalase activity was decreased by 1.4-fold (P < 0.05) next day after ceftriaxone withdrawal, but was increased between 14^th^ and 56^th^ days after antibiotic withdrawal (Fig 5C).

**Fig 6:**
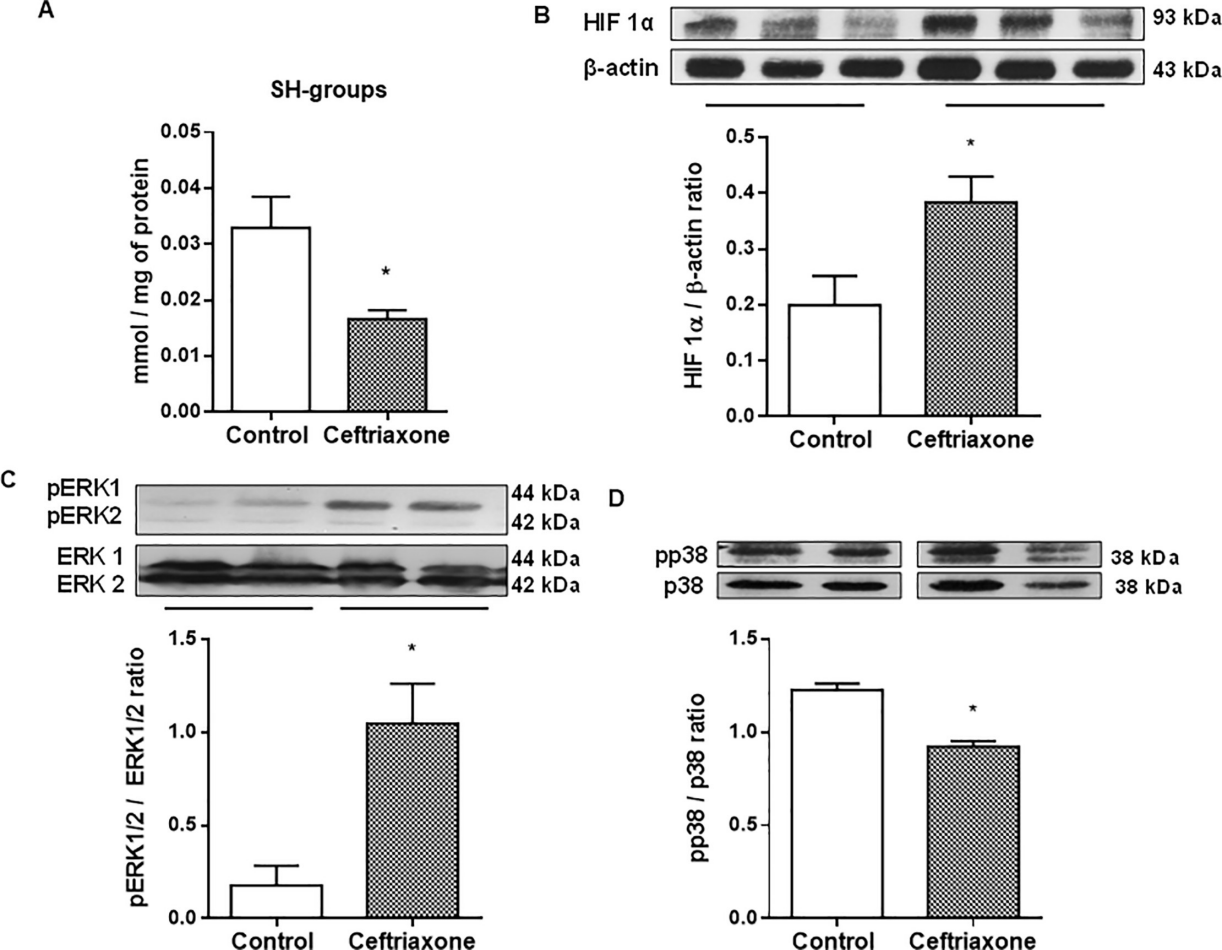
Protein SH-groups oxidation, HIF1α redox-sensitive transcription factor activation, and Erk 1/2 MAP kinases phosphorylation in colonic mucosa after ceftriaxone administration (300 mg/kg, i.m., 14 days). (A) Level of protein SH-groups in colonic mucosa 1 day after ceftriaxone withdrawal. (B) HIF1α in colonic mucosa 1 day after ceftriaxone withdrawal. (C) ERK1/2 phosphorylation (pERK /ERK ratio) in colonic mucosa 1 day after ceftriaxone withdrawal. (D) p38 phosphorylation (pp38/p38 ratio) in colonic mucosa 1 day after ceftriaxone withdrawal. Proteins levels in colonic mucosa were determined by Western blot. 1 –control, 2 –ceftriaxone group; n = 30; M ± SEM; * P < 0.05 *vs*. control values.

Oxidant-antioxidant balance disturbance can activate different redox-sensitive signaling pathways by oxidizing cysteine SH-groups of protein molecules. We detected a 1.9-fold (P < 0.05) decrease of the level of reduced protein SH groups (Fig 6A) with concomitant 1.9-fold (P < 0.05) increase of the level of hypoxia-sensitive transcription factor HIF1α next day after ceftriaxone withdrawal (Fig 6B). These changes were accompanied by an increase in ERK1/2 (Fig 6C) and decrease of p38 MAP kinases phosphorylation (Fig 6D) in colonic mucosa.

Thus, ceftriaxone-induced disturbance in colonic SCFAs system evoked the oxidant-antioxidant disbalance in colonic mucosa, hypoxia and activation of ERK1/2 MAP-kinases accompanied by inhibition of p38 MAP-kinases.

### A 2-week ceftriaxone treatment provoked the delayed intestinal barrier disruption

**Fig 7:**
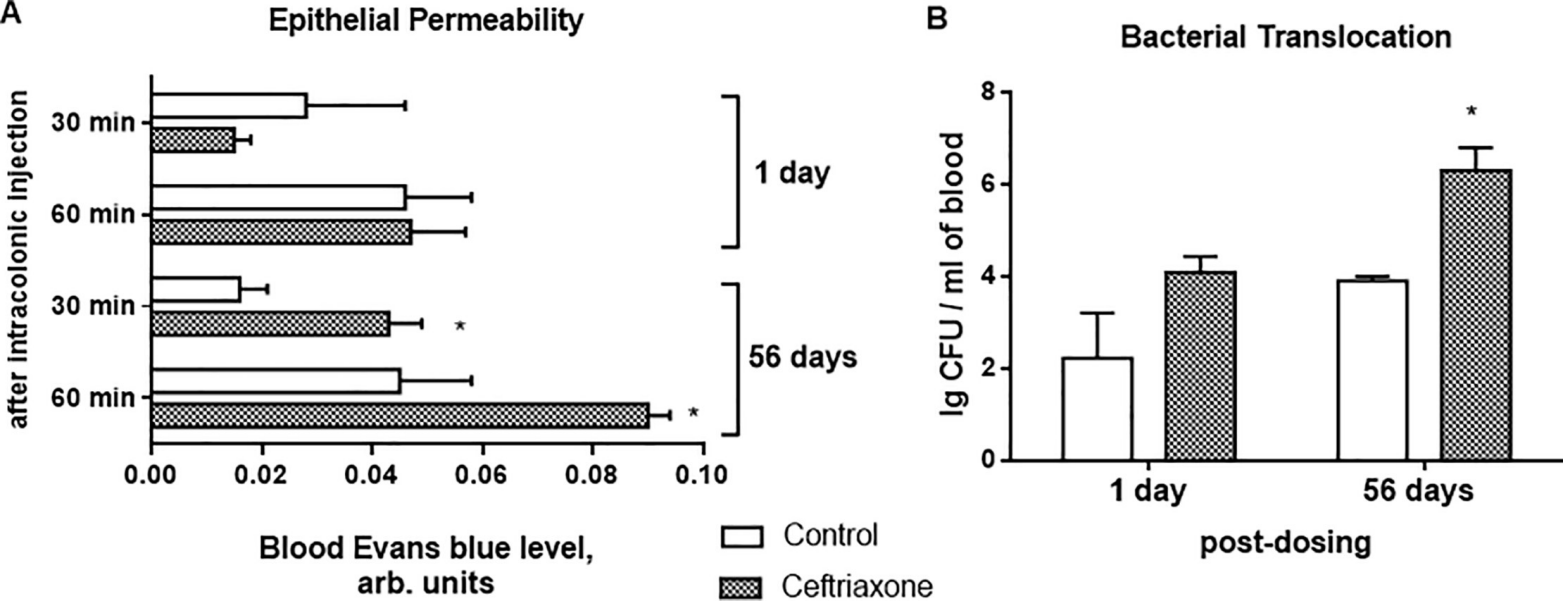
Colonic epithelial permeability was increased 56 days after ceftriaxone (300 mg/kg, i.m., 14 days) withdrawal. Evans blue permeation from the colonic lumen to the blood for 30 and 60 min in urethane-anesthetized rats 1 day and 56 days (A) after ceftriaxone withdrawal; (B) Bacteria load in rats blood collected from the portal vein 1 day and 56 days after ceftriaxone withdrawal; n = 16; M ± SEM; * P < 0.05 *vs*. control values.

Since the intestinal barrier is the main physical barrier, which is essential for prevention of intestinal inflammation development, next we assessed colonic epithelial permeability with the Evans blue permeation method. Next day after ceftriaxone withdrawal, we did not observe any significant changes in colonic epithelial permeability (Fig 7A). At 56 days after ceftriaxone withdrawal, Evans blue amount, which penetrated into the blood for 30 min and 60 min increased 2.7-fold and 2-fold (P < 0.05), respectively in ceftriaxone-treated rats in comparison to control group. This was also accompanied by 1.6 fold (P < 0.05) increase in the number of bacteria in the blood of the portal vein in the ceftriaxone-treated rats (Fig 7B).

**Fig 8:**
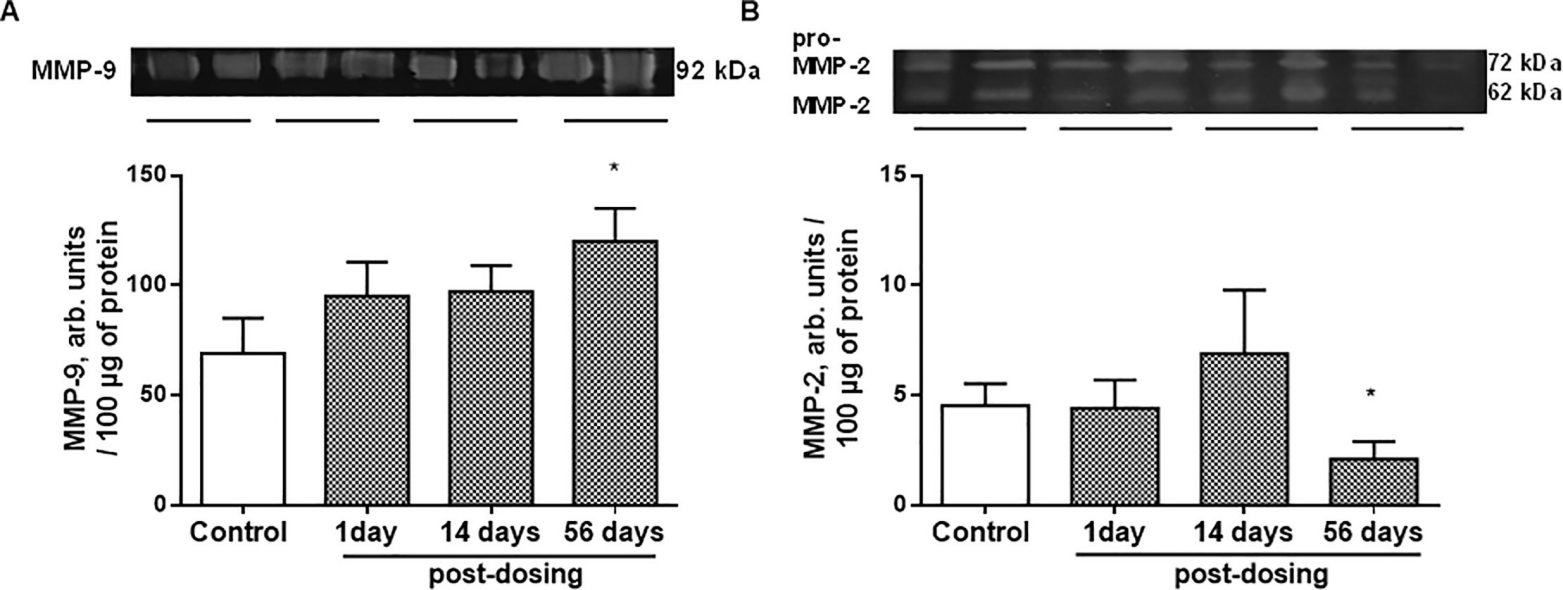
**Gelatinase activity of matrix metalloproteinase (MMP)-9 (A) and MMP-2 (B) in rat colonic mucosa 1, 14, 56 days after ceftriaxone (300 mg/kg, i.m., 14 days) withdrawal.** Activity was examined by electrophoretic zymography; n = 16; M ± SEM; * P < 0.05 *vs*. control values.

Moreover, the intestinal homeostasis disturbance was further confirmed by an increased proteolytic activity of matrix metalloproteinase (MMP) -9 (Fig 8A) and decreased–MMP-2 (Fig 8B).

**Fig 9:**
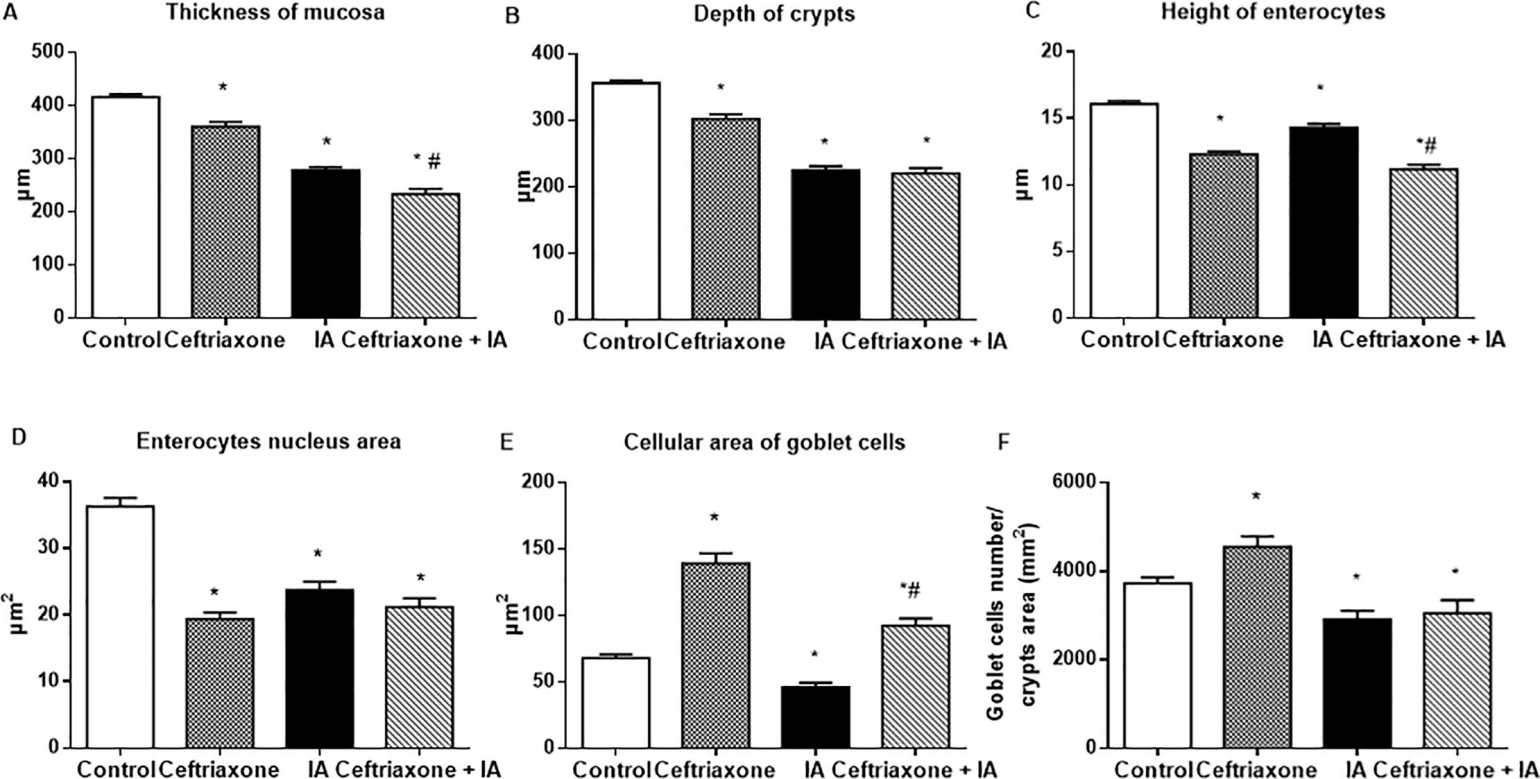
**Morphometric analysis of colonic mucosa in rats 56 days after ceftriaxone withdrawal and during iodoacetamide (IA)-induced colitis in rats**: thickness of mucosa (A), depth of crypts (B), height of enterocytes (C) and area of enterocytes nucleus per section (D), cellular area of goblet cell per section (E) and goblet cells number per crypts area (F). Control–rats, receiving sterile water for 14 days (1-14^th^ days) and methylcellulose vehicle 56 days after ceftriaxone withdrawal; IA–sterile water for 14 days (1-14^th^ days) and 3% IA 56 days after ceftriaxone withdrawal; Ceftriaxone + IA–ceftriaxone (1-14^th^ days) and 3% IA 56 days after ceftriaxone withdrawal. M ± SEM; n = 15; * P<0.05 *vs*. control values; # P<0.05 *vs*. IA group.

Microscopically, 56 days after ceftriaxone withdrawal, we observed decrease in the thickness of colonic mucosa (1.2-fold, P < 0.05), depth of crypts, height of enterocytes (1.3-fold, P < 0.05), and area of enterocytes nucleus (1.9-fold, P < 0.05) compared with control values (Fig 9A–9D) and goblet cells hypertrophy. The cellular area of PAS-positive goblet cells increased 2-fold and the number of goblet cells– 1.2-fold compared with control values (P < 0.05) (Fig 9E and 9F).

These results indicate that antibiotic-induced long-term changes in microbiota metabolic profile are associated with morphological remodeling of intestinal barrier and increased epithelial permeability, which is the feature of intestinal inflammation.

### A 2-week ceftriaxone treatment increased susceptibility to iodoacetamide-induced colitis in rats

To further confirm the predisposition of antibiotic-treated animals to the development of colonic inflammation long-after therapy, rats were intracolonically injected with 3% alkylating agent iodoacetamide (IA) 56 days after ceftriaxone or placebo administration.

**Fig 10:**
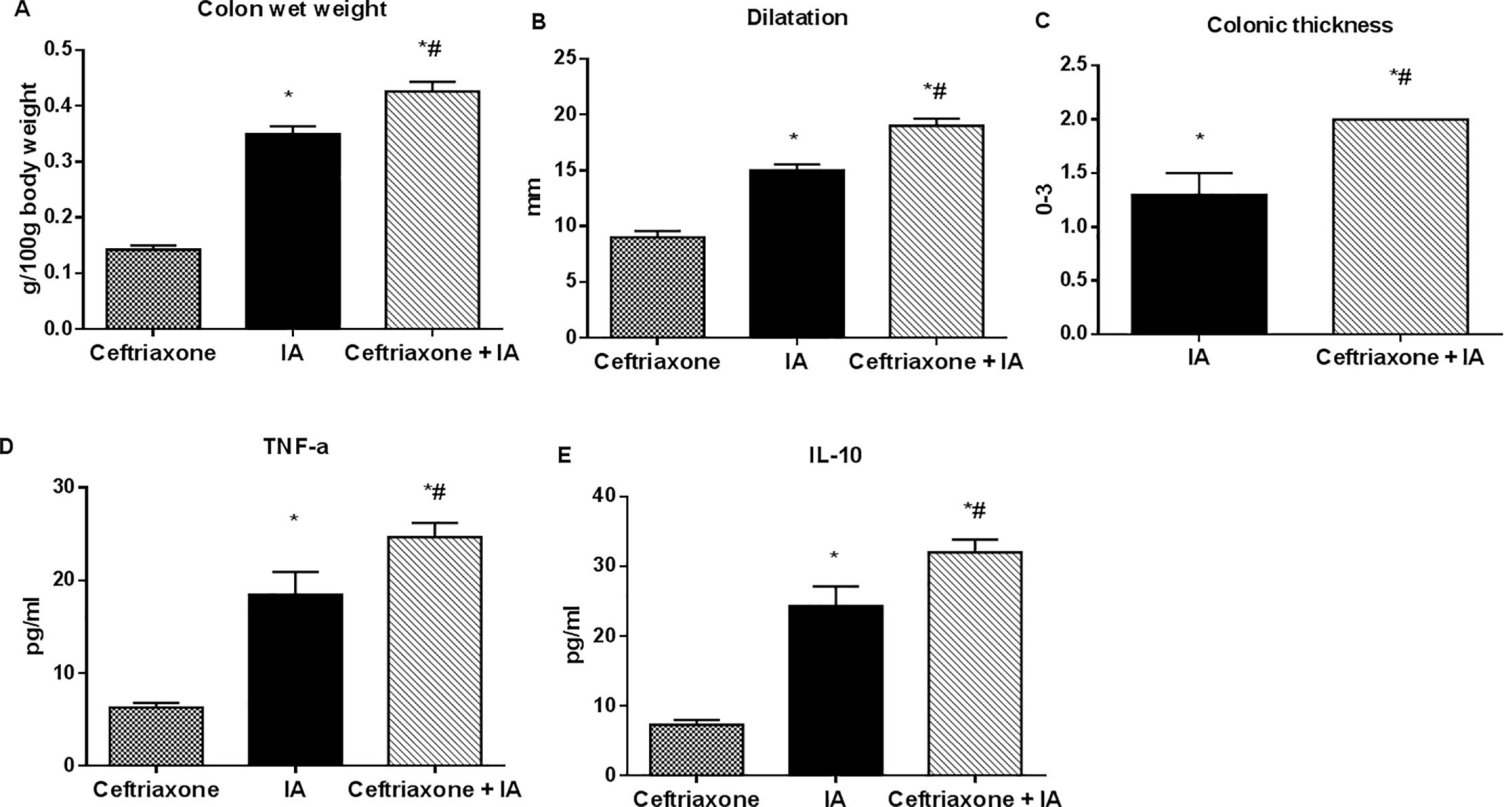
Ceftriaxone pretreatment increased edema (signs of inflammation) in colonic tissue during iodoacetamide (IA)-induced colitis in rats. The effects of ceftriaxone pretreatment for 14 days (300 mg/kg, i.m.) on macroscopic features of experimental colitis induced in rats by 3% IA (0.1 ml per rectum) 56 days after antibiotic withdrawal, autopsy at 6 h after IA enema: colon wet weight (A), colonic dilatation (B), colonic thickness (C). Ceftriaxone pretreatment increased serum levels of TNF-α (D) and IL-10 (E) during IA-induced colitis in rats. Control–rats, receiving sterile water for 14 days (1-14^th^ days) and methylcellulose vehicle 56 days after ceftriaxone withdrawal; IA–sterile water for 14 days (1-14^th^ days) and 3% iodoacetamide 56 days after ceftriaxone withdrawal; Ceftriaxone+ IA–ceftriaxone (1-14^th^ days) and 3% iodoacetamide 56 days after ceftriaxone withdrawal. M ± SEM; n = 15; * P<0.05 *vs*. control values; # P<0.05 *vs*. IA group.

As shown in Fig 10, ceftriaxone pretreatment markedly increased signs of IA-induced colitis. At 6 h after IA enema the ratio of colon wet weight/100 g of the body weight was increased 1.2 fold (P < 0.05), colonic dilatation– 1.1 fold (P < 0.05) and colonic thickness– 1.5 fold (P < 0.05) *vs*. placebo pretreated group.

Microscopically, the colon of IA-treated rats was characterized by signs of colitis development. Briefly, mucosal destruction and surface ulceration, crypt abscess and submucosal edema, goblet cells depletion were observed. The thickness of mucosa and depth of crypts, the height of enterocytes and area of enterocytes nucleus were significantly reduced, indicating atrophic processes (Fig 9).

Ceftriaxone pretreatment increased histopathological signs of the IA-induced colitis in rats. This was characterized by 1.2 fold (P < 0.05) decrease thickness of colonic mucosa and 1.3 fold (P < 0.05) decrease height of enterocytes in the ceftriaxone-pretreated group (Ceftriaxone + IA) compared with colitis group (IA) (Fig 9A and 9C). There was a tendency to decrease depth of crypts and area of enterocytes nucleus, although these changes were not statistically significant. The induction of experimental colitis in ceftriaxone-pretreated rats also led to expanding in the cellular area of goblet cells (Fig 9E). However, considering the fact that ceftriaxone administration alone induced goblet cells hypertrophy in colon these changes do not seem to be an increase from normal state as reducing from the considerably hypertrophic state.

Also, serum levels of both the proinflammatory cytokine TNF-α and anti-inflammatory IL-10 were higher (1.3-fold, P = 0.05) in ceftriaxone-pretreated rats *vs*. colitis group without antibiotic treatment (Fig 10D and 10E). Thus an overall enhancement in inflammation after antibiotic administration is attributable to increased inflammatory signaling.

## Discussion

The present study is the first report on the role of the SCFA system in the long lasting side effects of antibiotic treatment and its implication in IBD development.

There is increasing evidence that antibiotic-associated disturbance of gut microbiota might be persistent [19–20] and affect the immune tolerance and pathogen sensitivity of the gastrointestinal tract for a long time that contributes to the development of inflammatory diseases [24–25, 27, 43–44]. It was shown that oral administration of cephalosporin antibiotic cefoperazone induces disturbance of the cecum microbiota in mice 6 weeks after antibiotic withdrawal [45], oral administration of clinically recommended doses of clindamycin to healthy volunteers for 7 days induced sustained violations of the intestinal microbiota within the next 2 years [46], administration of ciprofloxacin or clindamycin over a period of 10 days caused changes in fecal microbiota that occurred within 12 months after drug administration [19].

All attempts to reveal the mechanism underlying the antibiotic-associated predisposition to IBD development were: (*i*) designed to induce dysbiosis with cocktail of antibiotics (which does not represent a clinically relevant situation) [26, 47]; (*ii*) the analyses of antibiotic-induced effects performed short-term after withdrawal of antibiotic therapy (that did not represent the mechanism of any long-term effects) [26–27]; (*iii*) there was indirect effect of antibiotic in the study on offsprings from antibiotic-treated mother [48].

The present study has been done on adult rats treated with clinically relevant dose of single broad-spectrum antibiotic ceftriaxone and analyses have been performed at 1, 14 and 56 days after antibiotic withdrawal. Similar to other studies [19, 45–46, 48] we found sustained changes in microbiota composition even at 56 days after ceftriaxone withdrawal. These changes were accompanied by an increase of cecum weight in antibiotic-treated rats, which is marker of dysbiosys [42, 49] and as a result the disturbance of fermentation process in colon [15].

Short-chain fatty acids (SCFAs) are main end-products of anaerobic fermentation of dietary fiber by large intestine microbiota [15], which provide the major source of energy for colonocytes, exert anti-inflammatory effects and regulate the growth of known pathogens [50–51]. Therefore, their levels may reflect the metabolic activity of intestinal microbiota. There are several reports on immediate changes in SCFAs levels after antibiotic therapy [29, 31–32, 52].

We showed sustained decreased level of SCFAs in rat feces long-after ceftriaxone withdrawal. The most profound changes observed for propionic and butyric acids, which led to an increase in the relative amount of the acetic acid. These changes reflect disbalance between putatively pro-inflammatory properties of acetic acid [53] and the anti-inflammatory butyric and propionic acids [54].

Only 5–10% of the total amount of SCFAs is excreted via feces [15]. Absorption of SCFAs in the cecum and the colon is a very efficient process that involves both passive diffusion and carrier-mediated transport through Na^+^-dependent SMCT and proton-dependent MCT transporters [15]. At the physiological colonic luminal pH (5.6–6.6), the major form of SCFAs is the anionic form, not available for simple diffusion. Thus, it seems obvious that *in vivo* SCFAs transport in mammals involves a carrier-mediated process [15]. Our immunohistochemical study has shown localization of the MCT1 & MCT4 transporters on the basolateral membrane of enterocytes, while SMCT was restricted to the apical cell membrane, which is consistent with the results of the previous studies [55]. It suggests the involvement of SMCT1 in the uptake of luminal SCFAs, and MCT in the efflux of SCFAs towards circulation. The immunoreactivity of SMCT1 was significantly decreased after ceftriaxone administration. Cresci et al. [56] have shown that the expression of SMCT1 is reduced markedly in germ-free mice and returned to normal levels after colonization with bacteria. Moreover, SMCT1 expression is silenced in colon cancer in humans, in a mouse model of intestinal/colon cancer, and in colon cancer cell lines [57]. Since SMCT1 is expressed in the apical membrane, the presence of butyrate and other SCFAs in the intestinal lumen would facilitate Na^+^ and water absorption, which may have a protective effect against diarrheal disease. Our previous study reported that ceftriaxone administration resulted in a reduction of water absorption in the colon by about 30% [58]. Energy metabolism in the colon is unique in that colonocytes use SCFAs, particularly butyrate, as a primary energy source [59–60], whereas most other tissues in the body use glucose. Ceftriaxone administration increased the immunoreactivity of the MCTs in the rat distal colonic mucosa, which implies that colonocytes tried to compensate energy source from the bloodstream, in response to the loss of luminal SCFAs. Thus, our study confirmed that gut bacteria play an active role in the control of gene expression in the host intestinal tract, promoting the expression of the genes that are required for biological actions of the bacterial fermentation products.

The SCFAs mediate their effect via G-protein-coupled receptors (GPCRs), FFA3 and FFA2 [5–7]. Propionate is the most potent agonist for both FFA3 and FFA2. Acetate is more selective for FFA2, whereas butyrate is more active on FFA3 [6]. We revealed expression of FFA2 receptors on surface enterocytes and at the crypt bottom, while FFA3 receptors on surface enterocytes, goblet cells and neurons of myenteric ganglia in rat colonic tissue, which is consistent with the previous studies [61–63]. Immediatly after ceftriaxone treatment there was a significant reduction in the levels of both FFA2 and FFA3 receptors. However, at later time points there was remarkable reduction only in FFA2 levels. Markedly reduced or completely lost FFA2 immunoreactivity was observed in most colorectal adenocarcinoma tissues [64]. GPR43 (FFA2 receptors)-deficient (Gpr43−/−) mice showed exacerbated or unresolving inflammation in models of colitis, arthritis, asthma and gout [11–12, 65–66].

Metabolic disturbance of the colonic microbiota was accompanied by decreased antioxidant enzymes (SOD and catalase) activity with subsequent increase of the intensity of lipid peroxidation (MDA level) in colonic mucosa after ceftriaxone administration. Thus, antibiotics can lead to long-term oxidative disturbance in the colonic mucosa of rats. Similarly, nutrient-deficient and energy-deficient colonocytes of germ-free mice experienced alterations in their redox state because of inadequate butyrate availability and decreased cellular oxidative phosphorylation with a resultant increase in oxidative stress [50, 67]. Increasing oxygenation of the mucosal surface promotes the outgrowth of aerotolerant taxa such as mouse pathogen *Citrobacter rodentium* [68]. Also, it has been shown that oxidative stress is one of the main mechanisms involved in the pathogenesis of IBD [69–70].

Changes in the tissue redox state implicate activation of redox-sensitive transcriptional factors. Cysteine SH groups oxidation of the transcriptional factor protein molecule involves regulation of the activity of redox-sensitive transcription factors [71]. We found a 1.9-fold decrease of protein SH groups in colonic mucosa next day after ceftriaxone withdrawal. Our previous results showed an increase in the levels of redox-sensitive transcription factors Egr -1 and Sp-1 in the rat colonic mucosa after 5 days administration of ceftriaxone [72]. This study revealed 2-fold increased level of the HIF1α in colonic mucosa after ceftriaxone administration. Since inflammation and hypoxia are linked [73], our results imply the development of proinflammatory changes in the response to oxidant-antioxidant balance disturbance in colonic mucosa. Moreover, study on luminal epithelium revealed that hypoxia-increased HIF 1α, COX-2 levels positively correlated with upregulation of MCT1 and MCT4 mRNA expression [74].

To understand signal-transducing enzymes involved in these functional changes of the colonic mucosa, we examined extracellular signal-regulated (ERK) 1/2 and p38 mitogen-activated protein kinases (MAPK) which are involved in the intracellular transmission and interpretation of the signals of inflammation and stress [75]. Ceftriaxone administration increased Erk1/2 and decreased p38 MAP kinases activity in the colonic mucosa. It is known that ERK1/2 may be phosphorylated and therefore directly activated through high levels of ROS [76], such as xanthine oxidase-derived H_2_O_2_ [77]. SCFAs can also induce a time-dependent phosphorylation of ERK1/2 through stimulation of FFA2 and FFA3 receptors [6]. Mice with the intestinal epithelial cell (IEC)-specific p38α downregulation are more susceptible to colitis, which was associated with increased ERK1/2 and JNK signaling, intestinal permeability and reduced number of goblet cells [78–79]. More severe colitis observed in these mice was rescued by the administration of probiotics VSL#3, which restored the altered epithelial permeability [79].

Upon treatment with acetate or FFAR2- and FFAR3-specific synthetic agonists, human monocytes displayed elevated p38 phosphorylation and attenuated C5, CCL1, CCL2, GM-CSF, IL-1α, IL-1β and ICAM-1 inflammatory cytokine expression. Acetate and FFAR2 agonist treatment also suppressed Akt and ERK2 signaling [80].

Hypoxia and oxidative stress development with subsequent activation of redox-sensitive transcription factors are associated with the disturbance of the intestinal barrier integrity at the early stages of IBD [81]. In our previous study we found decreased surface mucus glycoproteins levels with changes in their carbohydrates composition in the rat colon after antibiotic therapy [82], which was typical for IBD [83–84]. In the present study, we found increased colonic epithelial permeability to Evans blue dye that was further confirmed by increased bacterial translocation from the lumen to the blood as late as 56 days after ceftriaxone withdrawal. Moreover, we revealed morphological remodeling of the colonic tissue, increased of MMP-9 and decreased MMP-2 activity in rat colonic tissue 56 days after antibiotic withdrawal. Epithelial-derived MMP-9 is an important mediator of tissue injury in colitis, whereas MMP-2 protects against tissue damage and maintains gut barrier function [85–86]. MMP-9 expressed by epithelial cells mediates inflammation during colitis by modulating cell-matrix interaction and wound healing [87] and simultaneous increasing in proinflammatory cytokine Kc [88]. As a consequence of impaired colonic mucosal barrier and epithelium function, dysbiotic gut microbiota may penetrate into mucosa leading to an enhanced immune response, and proneness to the inflammatory reaction development.

Our results have shown that ceftriaxone pretreatment significantly reinforces inflammation during experimental iodoacetamide-induced colitis 56 days after antibiotic withdrawal. It was confirmed by increased histopathology of colitis, goblet cell dysfunction, colonic dilatation and wall thickening, and increased serum levels of inflammatory cytokines (TNF-α and IL-10).

These findings suggest that antibiotic-induced dysbiosis of colon microbiota can modulate the immune response in the gut. Consistent with our findings a recent prospective observational study [89] reported that antibiotic exposure over the 4-year study period in patients with IBD for both IBD and non-IBD-related reasons is associated with the more severe clinical presentation. Antibiotic-exposed patients have an elevated rate of C-reactive protein compared with nonantibiotic-exposed patients, which indicates increased inflammation.

Taken together our results provide novel insight into the importance of the composition of the microbiota for overall health. We have found that administration of broad-spectrum antibiotic ceftriaxone disturbs host-microbe signaling with a profound effect on the colonic functional state long-term after antibiotic withdrawal. SCFAs receptors and transporters potentially provide a molecular link between gastrointestinal bacterial metabolism and colonic mucosa functional state. Therefore, these findings provide plausible mechanisms by which antibiotic treatment increases the risk of IBD development.

## Supporting information

S1 File All data points behind means. (DOC)
