# Supplementary material for: The long-term consequences of antibiotic therapy: Role of colonic short-chain fatty acids (SCFA) system and intestinal barrier integrity
Source: PLoS One. 2019 Aug 22;14(8):e0220642. doi: 10.1371/journal.pone.0220642 (PMC6705842; doi:10.1371/journal.pone.0220642)
Supplement: S1 File — (DOC) [file pone.0220642.s001.doc]

**Table 1. Weight of rat cecum at different time points after ceftriaxone withdrawal**

| Group | Cecal weight, % of body weight | | |
| --- | --- | --- | --- |
|  | 1 day | 14 days | 56 days |
| Control | 1.441 | 1.416 | 1.792 |
|  | 1.520 | 1.320 | 1.758 |
|  | 2.025 | 1.130 | 1.342 |
|  | 1.253 | 1.448 | 1.522 |
|  | 1.631 | 1.046 | 1.774 |
|  |  |  | 1.680 |
|  |  |  | 1.609 |
|  |  |  | 2.013 |
| Mean | **1.574** | **1.272** | **1.686** |
| SEM | **0.128** | **0.079** | **0.071** |
| Ceftriaxone | 4.181 | 1.495 | 2.224 |
|  | 5.347 | 2.176 | 1.382 |
|  | 5.038 | 1.743 | 1.995 |
|  | 3.977 | 2.209 | 1.626 |
|  | 4.578 | 2.145 | 2.435 |
|  |  |  | 2.116 |
|  |  |  | 1.854 |
|  |  |  | 2.649 |
|  |  |  | 2.718 |
|  |  |  | 2.512 |
|  |  |  | 2.170 |
|  |  |  | 2.011 |
| Mean | **4.624** | **1.954** | **2.141** |
| SEM | **0.256** | **0.143** | **0.116** |

**Table 2. Gastrointestinal transit time next day after ceftriaxone withdrawal**

| Control | | | | Ceftriaxone | | | |
| --- | --- | --- | --- | --- | --- | --- | --- |
| Rat | Start | Stop | Transite time,  min | Rat | Start | Stop | Transite time,  min |
| A | 9:10 | 15:35 | 385 | A | 9:15 | 18:35 | 560 |
| [B](../../../../) |  | 15:10 | 360 | [B](../../../../) |  | 19:20 | 605 |
| C |  | 13:40 | 270 | C |  | 19:25 | 610 |
| D |  | 17:35 | 505 | D |  | 19:15 | 600 |
| E |  | 16:15 | 425 | E |  | 16:20 | 425 |
| Mean | | | **389** | Mean | | | 560 |
| SEM | | | **38.6** | SEM | | | 34.9 |

**Table 3. The level of SCFAs in rat feces at different time points after ceftriaxone withdrawal**

| Group | SCFAs, µmol / g wet feces | | | |
| --- | --- | --- | --- | --- |
|  | Acetic | Propionic | Butyric | Total  (C2-C6) |
| Control | 5.812 | 3.118 | 1.850 | 10.986 |
|  | 6.145 | 3.942 | 2.520 | 12.606 |
|  | 5.096 | 3.145 | 1.180 | 9.421 |
|  | 16.070 | 11.569 | 2.678 | 30.317 |
|  | 15.520 | 8.356 | 4.835 | 29.064 |
|  | 7.127 | 7.249 | 4.199 | 18.772 |
|  | 22.231 | 17.509 | 8.058 | 47.798 |
|  | 6.928 | 4.185 | 1.294 | 12.406 |
|  | 11.024 | 6.736 | 4.301 | 25.848 |
|  | 13.805 | 7.411 | 6.537 | 29.844 |
| Mean | **10.976** | **7.322** | **3.745** | **22.706** |
| SEM | **1.817** | **1.413** | **0.722** | **3.839** |
| Ceftriaxone, 1 day | 5.212 | 0.688 | 0.636 | 6.536 |
|  | 4.380 | 0.756 | 0.511 | 5.646 |
|  | 1.782 | 0.148 | 0.170 | 2.101 |
| Mean | **3.791** | **0.531** | **0.439** | **4.752** |
| SEM | **1.033** | **0.192** | **0.139** | **1.364** |
| Ceftriaxone, 56 days | 9.442 | 2.497 | 3.859 | 16.762 |
|  | 7.161 | 2.106 | 4.199 | 13.466 |
|  | 8.193 | 2.619 | 3.689 | 15.184 |
| Mean | **8.265** | **2.407** | **3.916** | **15.137** |
| SEM | **0.660** | **0.155** | **0.150** | **0.952** |

**Table 4.** FFA2 and FFA3 receptors in rat colonic mucosa at 1, 14 and 56 days after ceftriaxone withdrawal

| Group | Receptor / b-actin ratio | | | |
| --- | --- | --- | --- | --- |
|  | Control | 1 day | 14 days | 56 days |
| **FFA2** | 0.43640 | 0.00974 | 0.08798 | 0.00229 |
|  | 0.02368 | 0.00306 | 0.00011 | 0.00178 |
| Mean | **0.23004** | **0.00640** | **0.04404** | **0.00204** |
| SEM | **0.20636** | **0.00334** | **0.04394** | **0.00026** |
| **FFA3** | 0.20040 | 0.00735 | 0.15442 | 0.23549 |
|  | 0.08031 | 0.02266 | 0.35745 | 0.15830 |
| Mean | **0.14036** | **0.01500** | **0.25593** | **0.19690** |
| SEM | **0.06005** | **0.00766** | **0.10152** | **0.03859** |

**Table 5.** Malondialdehyde (MDA) level and Superoxide Dismutase (SOD) and Catalase antioxidant enzymes activity after ceftriaxone dosing

| Group | MDA,  nmol/mg of protein | SOD,  arb. units / 100 µg of protein | Catalase,  µmol H2O2/min*mg of protein |
| --- | --- | --- | --- |
| Control | 33.94 | 37.36 | 13.28 |
|  | 44.71 | 39.61 | 13.24 |
|  | 42.95 |  | 12.72 |
|  | 124.96 |  | 8.99 |
|  | 60.81 |  | 12.57 |
|  | 68.44 |  | 9.82 |
|  | 97.20 |  | 10.69 |
|  | 159.57 |  | 17.25 |
|  | 88.02 |  | 18.85 |
|  | 97.74 |  | 12.34 |
|  | 175.79 |  | 12.67 |
| Mean | **90.37** | **38.48** | **12.95** |
| SEM | **14.23** | **1.12** | **0.88** |
| **Ceftriaxone, 1 day** | 349.44 | 13.98 | 7.93 |
|  | 281.08 | 29.56 | 8.71 |
|  | 420.42 |  | 9.37 |
|  | 424.32 |  | 10.36 |
| Mean | **368.8** | **21.77** | **9.09** |
| SEM | **33.93** | **7.79** | **0.52** |
| **Ceftriaxone, 14 days** | 309.08 | 21.66 | 16.61 |
|  | 352.92 | 18.37 | 19.29 |
|  | *73.365** |  | 14.91 |
|  | 126.49 |  | 12.92 |
|  |  |  | 16.19 |
| Mean | **262.8** | **20.01** | **15.98** |
| SEM | **69.34** | **1.64** | **1.05** |
| **Ceftriaxone, 56 days** | 140.84 | 19.83 | 20.97 |
|  | 215.89 | 19.91 | 21.35 |
|  | 102.49 |  | 15.72 |
|  | 177.58 |  | 26.71 |
|  | 262.25 |  | 12.51 |
|  | 102.50 |  | 15.35 |
| Mean | **166.9** | **19.87** | **18.77** |
| SEM | **26.19** | **0.51** | **2.12** |

**Table 6.** Level of protein SH-groups in colonic mucosa 1 day after ceftriaxone withdrawal

|  | SH-groups, mmol / mg of protein | |
| --- | --- | --- |
|  | **Control** | **Ceftriaxone** |
|  | 0.029 | 0.017 |
|  | 0.053 | 0.014 |
|  | 0.024 | 0.023 |
|  | 0.022 | 0.015 |
|  | 0.035 | 0.014 |
| Mean | **0.033** | **0.017** |
| SEM | **0.006** | **0.002** |

**Table 7.** HIF1α, Erk 1/2 & p38 phosphorylation in colonic mucosa 1 day after ceftriaxone withdrawal

| Group | **HIF 1a / b-actin ratio** | **pERK1/2 / ERK1/2 ratio** | **pp38 / p38 ratio** |
| --- | --- | --- | --- |
| Control | 0.270 | 0.072 | 1.189 |
|  | 0.231 | 0.284 | 1.262 |
|  | 0.096 |  |  |
| Mean | **0.199** | **0.178** | **1.226** |
| SEM | **0.053** | **0.106** | **0.036** |
| Ceftriaxone | 0.432 | 1.262 | 0.894 |
|  | 0.428 | 0.830 | 0.952 |
|  | 0.292 |  |  |
| Mean | **0.384** | **1.046** | **0.923** |
| SEM | **0.046** | **0.216** | **0.029** |

**Table 8.** Evans blue permeation from the colonic lumen to the blood for 30 and 60 min in urethane-anesthetized rats 1 day and 56 days after ceftriaxone withdrawal

| Group | Time after intracolonic injection | Blood Evans blue level,  arb. units (n=5) | |
| --- | --- | --- | --- |
|  |  | Mean | SEM |
| Control, 1 day | 30 min | 0.028 | 0.018 |
|  | 60 min | 0.046 | 0.012 |
| Ceftriaxone, 1 day | 30 min | 0.015 | 0.003 |
|  | 60 min | 0.047 | 0.01 |
| Control, 56 days | 30 min | 0.016 | 0.005 |
|  | 60 min | 0.045 | 0.013 |
| Ceftriaxone, 56 days | 30 min | 0.043 | 0.006 |
|  | 60 min | 0.090 | 0.004 |

**Table 9.** Bacteria load in rats blood collected from the portal vein 1 day and 56 days after ceftriaxone withdrawal

| Group | Time after ceftriaxone withdrawal | lg CFU / ml of blood | |
| --- | --- | --- | --- |
|  |  | Mean | SEM |
| Control | 1 day | 2.23 | 0.98 |
|  | 56 days | 3.91 | 0.10 |
| Ceftriaxone | 1 day | 4.10 | 0.34 |
|  | 56 days | 6.31 | 0.50 |

**Table 10.** Gelatinase activity of matrix metalloproteinase (MMP)-9and MMP-2 in rat colonic mucosa 1, 14, 56 days after ceftriaxone (300 mg/kg, i.m., 14 days) withdrawal

| MMP | arb. units / 100 µg of protein | | | |
| --- | --- | --- | --- | --- |
|  | Control | 1 day | 14 days | 56 days |
| **MMP-9** | 66.68 | 79.89 | 109.56 | 106.95 |
|  | 106.84 | 75.88 | 79.70 | 125.00 |
|  | 28.48 | 141.10 | 73.45 | 160.41 |
|  | 73.06 | 82.40 | 124.49 | 89.62 |
| Mean | **68.77** | **94.82** | **96.80** | **120.50** |
| SEM | **16.06** | **15.49** | **12.14** | **15.14** |
| **MMP-2** | 6.01 | 3.11 | 5.98 | 5.26 |
|  | 9.77 | 8.29 | 15.05 | 0.87 |
|  | 0.61 | 3.08 | 1.40 | 1.95 |
|  | 1.74 | 3.15 | 5.09 | 0.31 |
| Mean | **4.53** | **4.41** | **6.88** | **2.10** |
| SEM | **1.00** | **1.29** | **2.90** | **0.81** |

**Table 11.** Morphometric analysis of colonic mucosa in rats 56 days after ceftriaxone withdrawal and during iodoacetamide (IA)-induced colitis in rats

| Parameter | | **Control** | **Ceftriaxone** | **IA** | **Ceftriaxone + IA** |
| --- | --- | --- | --- | --- | --- |
| Thickness  of mucosa (µm) | Mean | 416 | 360 | 278 | 233 |
|  | SEM | 5 | 9 | 6 | 10 |
| Depth of crypts,  µm | Mean | 356 | 302 | 225 | 220 |
|  | SEM | 4 | 7 | 6 | 8 |
| Height of enterocytes,  µm | Mean | 16.1 | 12.3 | 14.3 | 11.2 |
|  | SEM | 0.2 | 0.2 | 0.3 | 0.3 |
| Enterocytes nucleus  area, µm^2^ | Mean | 36.3 | 19.4 | 23.7 | 21.2 |
|  | SEM | 1.3 | 1 | 1.3 | 1.3 |
| Cellular area of  goblet cells, µm^2^ | Mean | 67.9 | 139.1 | 46.1 | 92.3 |
|  | SEM | 2.9 | 7.7 | 3.3 | 5.4 |
| Goblet cells number/  crypts area (mm2) | Mean | 3729 | 4546 | 2906 | 3050 |
|  | SEM | 132 | 239 | 199 | 298 |

**Table 12.** The effects of ceftriaxone pretreatment for 14 days (300 mg/kg, i.m.) on macroscopic features of experimental colitis induced in rats by 3% IA (0.1 ml per rectum) 56 days after antibiotic withdrawal

| Parameter | **Ceftriaxone** | **IA** | **Ceftriaxone + IA** |
| --- | --- | --- | --- |
| Colon wet weight,  g/100g body weight | 0.157 | 0.328 | 0.388 |
|  | 0.134 | 0.338 | 0.387 |
|  | 0.136 | 0.404 | 0.448 |
|  |  | 0.346 | 0.474 |
|  |  | 0.332 | 0.434 |
| Mean | **0.142** | **0.349** | **0.426** |
| SEM | **0.008** | **0.014** | **0.017** |
| Dilatation, mm | 9 | 13 | 19 |
|  | 8 | 15 | 19 |
|  | 9 | 16 | 17 |
|  | 9 | 15 | 19 |
|  |  | 16 | 21 |
| Mean | **9** | **15** | **19** |
| SEM | **0.25** | **0.55** | **0.63** |
| Colonic thickness, (0-3) | 0 | 2 | 2 |
|  | 0 | 1 | 2 |
|  | 0 | 1.5 | 2 |
|  | 0 | 1 | 2 |
|  | 00 | 1 | 2 |
| Mean | **0** | **1.3** | **2** |
| SEM | **0** | **0.2** | **0** |

**Table 13.** Serum levels of TNF-α and IL-10 in rats during iodoacetamide (IA)-induced colitis

| Parameter | **Ceftriaxone** | **IA** | **Ceftriaxone + IA** |
| --- | --- | --- | --- |
| **TNF-a** | 5.4 | 27.3 | 25.4 |
|  | 5.6 | 14.8 | 25.9 |
|  | 7.6 | 13.4 | 23.1 |
|  | 6.6 | 18 | 29.1 |
|  |  | 18.9 | 19.9 |
| Mean | **6.3** | **18.5** | **24.7** |
| SEM | **0.5** | **2.4** | **1.5** |
| **IL-10** | 6.2 | 34.6 | 32.5 |
|  | 5.9 | 19.7 | 34.1 |
|  | 8.8 | 18.5 | 29.9 |
|  | 8.2 | 23.9 | 37.2 |
|  |  | 24.9 | 26.3 |
| Mean | **7.3** | **24.3** | **32.0** |
| SEM | **0.7** | **2.8** | **1.9** |
